# Diagnostic validity and triage concordance of a physiotherapist compared to physicians’ diagnoses for common knee disorders

**DOI:** 10.1186/s12891-017-1799-3

**Published:** 2017-11-14

**Authors:** S. Décary, M. Fallaha, B. Pelletier, P. Frémont, J. Martel-Pelletier, J.-P. Pelletier, D. E. Feldman, M.-P. Sylvestre, P.-A. Vendittoli, F. Desmeules

**Affiliations:** 1 School of Rehabilitation, Faculty of Medicine, University of Montreal, Montreal, QC Canada; 2 Orthopaedic Clinical Research Unit, Maisonneuve-Rosemont Hospital Research Center, Centre intégré universitaire de santé et de services sociaux de l’Est-de-l’Île-de-Montréal, Montreal, QC Canada; 3 Department of Surgery, Maisonneuve-Rosemont Hospital, University of Montreal, Montreal, QC, Canada. Centre intégré universitaire de santé et de services sociaux de l’Est-de-l’Île-de-Montréal, Montreal, QC Canada; 4 Department of Rehabilitation, Faculty of Medicine, Laval University, Quebec City, QC Canada; 5 Osteoarthritis Research Unit, University of Montreal Hospital Research Center (CRCHUM), Montreal, QC Canada; 6 Department of Social Preventive Medicine, School of Public Health, Université de Montréal, Montreal, QC Canada

**Keywords:** Diagnosis, Knee disorders, Physiotherapist

## Abstract

**Background:**

Emergence of more autonomous roles for physiotherapists warrants more evidence regarding their diagnostic capabilities. Therefore, we aimed to evaluate diagnostic and surgical triage concordance between a physiotherapist and expert physicians and to assess the diagnostic validity of the physiotherapist’s musculoskeletal examination (ME) without imaging.

**Methods:**

This is a prospective diagnostic study where 179 consecutive participants consulting for any knee complaint were independently diagnosed and triaged by two evaluators: a physiotherapist and one expert physician (orthopaedic surgeons or sport medicine physicians). The physiotherapist completed only a ME, while the physicians also had access to imaging to make their diagnosis. Raw agreement proportions and Cohen’s kappa (k) were calculated to assess inter-rater agreement. Sensitivity (Se) and specificity (Sp), as well as positive and negative likelihood ratios (LR+/−) were calculated to assess the validity of the ME compared to the physicians’ composite diagnosis.

**Results:**

Primary knee diagnoses included anterior cruciate ligament injury (*n* = 8), meniscal injury (*n* = 36), patellofemoral pain (*n* = 45) and osteoarthritis (*n* = 79). Diagnostic inter-rater agreement between the physiotherapist and physicians was high (k = 0.89; 95% CI:0.83–0.94). Inter-rater agreement for triage recommendations of surgical candidates was good (k = 0.73; 95% CI:0.60–0.86). Se and Sp of the physiotherapist’s ME ranged from 82.0 to 100.0% and 96.0 to 100.0% respectively and LR+/− ranged from 23.2 to 30.5 and from 0.03 to 0.09 respectively.

**Conclusions:**

There was high diagnostic agreement and good triage concordance between the physiotherapist and physicians. The ME without imaging may be sufficient to diagnose or exclude common knee disorders for a large proportion of patients. Replication in a larger study will be required as well as further assessment of innovative multidisciplinary care trajectories to improve care of patients with common musculoskeletal disorders.

**Electronic supplementary material:**

The online version of this article (10.1186/s12891-017-1799-3) contains supplementary material, which is available to authorized users.

## Background

Knee disorders are a common reason for seeking diagnosis and management in primary care and can significantly impact quality of life of individuals [1–3]. However, evidence shows the limited ability of medical providers to perform an appropriate physical examination to make a diagnosis [4, 5]. This has led to an overreliance on imaging or inappropriate referral to specialists to confirm a diagnosis, which incurs increasing health care costs and unnecessary delays to initiate conservative care [4–8]. Models of care in which physiotherapists act as first contact providers have been proposed [4, 9–11]. In these models, physiotherapists act as consultants who evaluate the patient, make a diagnosis and offer conservative care or refer to other providers [4, 9–11].

To adequately take on these autonomous roles, physiotherapists need to be able to provide a valid clinical diagnostic impression and be able to refer accurately patients to other providers or surgical candidates to orthopaedic surgeons; they need do this in manner that is as effective as physicians with expertise in musculoskeletal disorders would do [1]. *Moore* et al. demonstrated the equivalence between physiotherapists and orthopaedic surgeons for the clinical diagnosis of common musculoskeletal disorders [5]. When compared to magnetic resonance imaging (MRI) results, the diagnostic agreement of the physiotherapists was almost as high as the orthopaedic surgeons (raw agreement: 74.5% compared to 80.8%), and it was superior to non-orthopaedic providers such as primary care physicians (35.4%) [5]. A systematic review reported that, based on moderate quality studies, inter-rater agreement kappa values ranged from 0.69 to 1.00 for diagnostic agreement between physiotherapists and orthopaedic surgeons and kappa values ranging from 0.52 to 0.70 for the triage of surgical candidates, indicating moderate to high agreement between providers [12].

However, in many primary care settings, imaging may be difficult to obtain rapidly or physiotherapists may not be allowed to order imaging and must therefore rely exclusively on musculoskeletal examination (ME) when assessing patients. *Jackson* et al. concluded in a meta-analysis including 35 diagnostic studies, not specific to physiotherapists, that a complete ME demonstrates adequate validity to include or exclude common knee disorders when compared to imaging or arthroscopic findings [1, 13]. However, it is not known whether this also applies to physiotherapists.

Therefore, the objectives of this study were to 1) evaluate agreement on the diagnosis and surgical triage between a physiotherapist using a standardized ME without the use of imaging results and physicians and 2) to assess the validity of the physiotherapist’s ME to diagnose common knee disorders.

## Methods

### Study design and settings

This study is part of a larger multi-center prospective diagnostic cohort study that aims to identify the optimal combination of elements from the history and physical examination for the diagnosis of common knee disorders. Recruitment took place in an outpatient orthopaedic clinic and a primary care family medicine clinic. All consecutive patients consulting one of the participating physicians for a new knee complaint between November 2014 and January 2016 were recruited. Also, we included participants from a university community (students, teaching staff and other personnel) if they sought a diagnosis and care for a current knee complaint. These participants received an email invitation to participate from September 2015 to January 2016. The present study, its design, methodology and reporting of results is based on the Standards for Reporting Diagnostic Accuracy Studies 2015 (STARD) [14, 15]. The study was approved by the hospital’s ethics committee. The study was explained by the physiotherapist to all participants and written informed consent was obtained from them prior to consultation.

### Participants

Inclusion criteria were: 18 years of age or older, consulting for a knee complaint for which they sought diagnosis, and being able to understand and speak French. Patients previously diagnosed and treated by one of the participating physicians were excluded to ensure that the patient did not reveal their previous diagnosis to the physiotherapist. We also excluded patients who had undergone lower limb surgery in the past six months, patients with a knee arthroplasty or who presented with more than two lower limb pathologies in addition to the one for which they were consulting or if they were diagnosed with any systemic inflammatory disorder.

### Data collection procedure

#### Patients’ characteristics and history elements

All clinical settings had the same data collection procedure. Upon arrival at the clinic, participants answered a questionnaire which included age, sex and anthropometric data (weight and height) to allow calculation of body mass index (BMI), duration of symptoms, history of the lesion (traumatic or non-traumatic), and presence of bilateral knee pain. Participants also completed the Knee Injury and Osteoarthritis Outcome Score (KOOS), a validated 42-item self-report questionnaire that assesses pain, symptoms, function in daily living, function in sport and recreation and knee-related quality of life [16–18]. Psychological distress was assessed using the Kessler-6 screening scale for serious mental disorders [19, 20].

#### Physical examination

Each participant was then independently assessed by two evaluators: a physiotherapist and one of the four physicians. The two evaluations were completed on the same day with a fifteen-minute interval between each. The physiotherapist always evaluated the participants prior to the physicians in a separate room. Both the physiotherapist and the physicians were blinded to each other results. Following the physiotherapist’s ME, the patients’ pain was evaluated using a three-point likert scale (light, moderate, severe) and they were withdrawn from the study if their pain was moderately or severely increased compared to the start of the evaluation. The physician then proceeded to his independent history taking and physical examination.

#### Diagnosis, reference standard and triage options

After independently seeing the patient, the physiotherapist and the physician each completed a separate form where they indicated their primary and, when applicable secondary diagnosis. The physiotherapist was blinded to imaging results and therefore determined his diagnosis on the sole basis of his ME [21].

As the reference standard, the physicians had access to imaging to establish their diagnosis and they performed their own analysis of the relevant imaging results. All participants were required to have a radiograph of their knee that included the following three views: weight-bearing antero-posterior view with lateral and skyline views [22]. Magnetic resonance imaging (MRI) was required when the physician suspected a ligament injury, a meniscal injury or any other uncertain diagnoses. If participants already had recent radiographs within 3 months of their participation or MRI results within 6 months with suitable views or scans that allow adequate interpretation and grading by the physician, these results were used. If the physician doubted that the imaging did not reflect the current stage of pathology another test was ordered. The physician made a final primary and secondary (if necessary) composite diagnosis based on the patient’s history, physical tests and imaging results [23]. This final composite diagnosis was considered the reference standard against which the physiotherapist’s diagnoses were compared to all participants. We also compared the physiotherapist’s diagnoses to the imaging diagnoses only as a secondary reference standard [24–27].

Lastly, both the physiotherapist and physicians independently selected the triage option - conservative, surgical or undecided - for the patients. For patients seen by the sports medicine physicians in the family medicine unit or from the university community, the patients were considered surgical cases if the physician considered that all other options would not be adequate and that requesting a surgical consultation was the proper conduct.

#### Standardisation and evaluators’ experience

Before the start of the study, physicians met with the research personnel to familiarize with the study protocol and verify if their usual practice differed from the other evaluators to improve concordance. All practitioners participated in the standardization of the techniques, interpretation of the physical tests and definition of the related diagnoses and all agreed to comply with the proposed definitions during their respective evaluation. The physiotherapist had one year of clinical experience. The four participating physicians (two orthopaedic surgeons and two sports medicine physicians [1]) each had more than 20 years of experience in the diagnosis and management of knee disorders.

### Sample size

We calculated the sample size to detect an overall inter-rater Kappa value for the overall diagnostic concordance greater than 0.80 assuming a two-tailed null hypothesis for a Kappa equal to 0.4 or less [28–30]. We estimated the proportions of positives agreement for knee disorders diagnoses and expected Kappa values based on a previous cohort from a similar setting recuited by our team [8]; the required sample size is set at 71 patients considering a 80% power [28–30].

### Statistical analysis

We used descriptive statistics to present the participants’ characteristics. All primary diagnoses were classified using five common categories: 1- ACL injury; 2- meniscal injury; 3- patellofemoral pain; 4- osteoarthritis; 5- others [31, 32]. In the event where the physiotherapist and the physician disagreed on the primary diagnosis, the secondary diagnoses were taken into account to further evaluate diagnostic concordance. To measure the inter-rater agreement for the diagnostic categories and triage recommendations between the physiotherapist and the physicians, proportions of raw agreement and Cohen’s Kappas with associated 95% confidence intervals (CI) were calculated. Because Kappa values may become biased due to high or low prevalence of concordant cases compared or non-concordant cases, bias index and prevalence index were calculated where 0 indicates no bias and 1 a high bias [31, 33]. *Prevalence and Bias Adjusted Kappas* (PABAK) were calculated for each diagnostic category to correct for these potential biases [31, 33]. Interpretation of inter-rater agreement was made according to the Landis and Koch scale in which 0 indicates poor agreement, 0–0.2 slight, 0.2–0.4 fair, 0.4–0.6 moderate, 0.6–0.8 substantial or good and >0.8 almost perfect or high agreement [28, 31]. For the validity of the ME, we compared the final diagnosis proposed by the physiotherapists’ ME with the physicians’ composite final diagnosis (reference standard) based on the ME, radiographs and MRI results when needed. Sensitivity (Se), specificity (Sp) and likelihood ratios with 95% CIs were calculated [34, 35]. Se and Sp relate respectively to the proportion of true positives and true negatives when a test is performed [36, 37]. Positive and negative likelihood ratios were used to evaluate the diagnostic validity of the physiotherapist’s physical examination compared to the physicians’ composite diagnosis and the following cut-offs were used: 1-to include a disorder a LR+ ≥5 and 2- to exclude a disorder a LR- ≤0.2 as they are reported to produce at least a moderate shift in post-test probability of having or not a certain disorder [37, 38]. Analysis was performed using SPSS version 21 (SPSS Inc., Chicago) and R version 3.2.3 (packages *epiR, irr* and *psych*, http://cran.r-project.org/).

## Results

Table 1 presents the characteristics of participants. Out of 198 eligible patients, five (2.5%) refused to participate, 14 (7.1%) were excluded before consultation and 179 (90.4%) were included in the study (see Additional file 1: Appendix 1). None were excluded following the physiotherapist’s evaluation because of increased pain or for any reasons. Mean age was 49.9±16.1 years old and most participants were female (63.7%) with a mean BMI of 29.1± 6.5 kg/m^2^. The majority of participants were recruited from the orthopaedic clinic (79.3%) and consulted for a non-traumatic disorder (73.7%). Most participants had pain for over 3 months at the time of consultation (90.5%). KOOS *Sports* and *Quality of life* domains were most severely affected (31.4±24.8 and 40.9±20.3).

**Table 1:** Characteristics of participants (*n* = 179)

Characteristics	n (%)	mean (SD)
Age (years)		49.9 (16.1)
Sex		
Female	114 (64)	
Male	65 (36)	
Body Mass Index (Kg\m^2^)		29.1 (6.5)
Recruitment site		
Orthopaedic clinic	142 (80)	
Family medicine unit	15 (8)	
University community	22 (12)	
History of trauma	47 (26)	
Bilateral knee pain	39 (22)	
Duration of pain at time of consultation		
<3 months	17 (10)	
3–12 months	45 (25)	
≥ 12 months	117 (65)	
KOOS- Knee Injury and Osteoarthritis Outcome Score (%)		
Pain		58.6 (19.7)
Symptoms		71.0 (19.6)
Activity of Daily Living		66.1 (21.8)
Sports		31.4 (24.8)
Quality of Life		40.9 (20.3)
K6 psychological distress scale (/30)		26.0 (4.5)

*SD* standard deviation; KOOS: 0 indicates a severe condition and 100 indicates a normal knee; K6: 6 indicates serious mental illness and 30 indicates no mental illness

Primary clinical diagnoses made by the participating physicians (using ME and imaging) included: anterior cruciate ligament injury (ACL) (*n* = 8), meniscal injury (*n* = 36), patellofemoral pain (PFP) (*n* = 45), osteoarthritis (OA) (*n* = 79) and other diagnoses (*n* = 11) (Table 2). All participants (*n* = 179) had radiograph results and 70 participants had an MRI scan. Based on imaging results only, diagnoses included: OA (*n* = 96), meniscal tears (*n* = 54), ACL tears (*n* = 16) or others (*n* = 5).

**Table 2:** Clinical and imaging diagnoses of participants (*n* = 179)

	n (%)
Primary clinical composite diagnoses	
Anterior cruciate ligament injury	8 (5)
Meniscal injury	36 (20)
Patellofemoral Pain Syndrome	45 (25)
Osteoarthritis	79 (44)
Other knee diagnoses	11 (6)
Imaging findings and diagnoses	
Osteoarthritis	96 (56)
K-L Grade 1	14 (15)
K-L Grade 2	36 (38)
K-L Grade 3	19 (20)
K-L Grade 4	13 (14)
Meniscal tears (n)	54 (32)
Medial meniscus	49 (91)
Lateral meniscus	8 (15)
Anterior cruciate ligament tears	16 (9)
Complete	6 (37)
Partial	8 (50)
Unclear	2 (13)
Posterior cruciate ligament tear	1 (1)
Soleus tear	1 (1)
Hamstring tendinopathy	2 (1)
Medial collateral ligament tear	1 (1)

*SD* standard deviation; Clinical diagnoses are composite diagnoses made by physicians using both musculoskeletal examination and imaging; Others knee diagnoses included: contusion of the tibial plateau (*n* = 2), PCL tear (*n* = 1), soleus tear (*n* = 1), psychosomatic origin (*n* = 1), muscular spasms linked to multiple sclerosis (*n* = 1), hamstring tendinopathy (*n* = 3), medial collateral ligament injury (*n* = 1), functional instability without meniscal or ACL injury (*n* = 1); Imaging diagnoses are based on imaging studies using radiograph or magnetic resonance imaging; Grades are for Kellgren-Lawrence scale in the most affected compartment; Radiographic OA was defined as K-L≥ 1

Table 3 presents the concordance between the diagnosis made by the physiotherapist using only the ME and the composite diagnosis made by physicians using both ME and imaging or with the imaging diagnoses only. The overall raw agreement between the physiotherapist and the physicians’ diagnosis was 92.2% with an high inter-rater agreement (κ=0.89, 95% CI: 0.83–0.94). Inter-rater agreement for specific knee disorders ranged from κ= 0.88 to 0.94. ACL injury and other diagnoses had fewer cases (8/179 and 11/179) which translated into a high prevalence index (0.91 and 0.89, respectively). However, all PABAK estimates were included in the Cohen’s kappa 95% confidence intervals and were therefore not significantly different, which indicates that even when bias were present (i.e: prevalence of ACL injuries), this did not influence the Kappa estimate [31]. When comparing the physiotherapists’ diagnosis with imaging only, raw agreement was slightly lower at 84.4% and inter-rater agreement was good (κ= 0.77; 95% CI: 0.68–0.85).

**Table 3:** Concordance between the physiotherapist and physicians’ composite or imaging only diagnoses (*n* = 179)

	Raw agreement	Cohen’s kappa	95% CI	Bias Index	Prevalence Index	PABAK
Overall concordance with the physicians’ composite diagnoses	92.2% (165/179)	0.89	0.83–0.94	–	–	–
ACL injury	100.0% (8/8)	0.94	0.82–1.00	0.01	0.91	0.99
Meniscal injury	97.2% (35/36)	0.88	0.80–0.93	0.03	0.57	0.92
Patellofemoral pain	91.1% (41/45)	0.88	0.80–0.96	0.05	0.50	0.91
Osteoarthritis	91.1% (72/79)	0.89	0.82–0.95	0.02	0.14	0.89
Other knee disorders	81.8% (9/11)	0.89	0.75–1.00	0.01	0.89	0.98
Overall concordance with imaging only diagnoses	84.4% (151/179)	0.77	0.68–0.85	–	–	–

*ACL* anterior cruciate ligament. 95% CI: 95% confidence interval. PABAK is only calculated only for 2 × 2 tables. Others knee diagnoses included: contusion of the tibial plateau (*n* = 2), PCL tear (*n* = 1), soleus tear (*n* = 1), psychosomatic origin (*n* = 1), muscular spasms linked to multiple sclerosis (*n* = 1), hamstring tendinopathy (*n* = 3), medial collateral ligament injury (*n* = 1), functional instability in the absence of ACL or meniscal injury (*n* = 1)

Table 4 presents the diagnostic validity of the physiotherapist’s standardized ME compared to the reference standard (physician’s composite diagnosis) to discriminate between each knee disorders. Sensitivity ranged from 82 to 100% and was lowest for *Others* knee disorders. Specificity ranged from 96 to 100%. Positive likelihood ratio ranged from 23.2 to 267.6 and all 95% CI lower bounds were above 10.0. Negative likelihood ratio ranged from 0.00 to 0.18 and all 95% CI upper bounds were below LR-≤ 0.20, except for *PFP* (LR- = 0.23) and *Others* (LR- = 0.65). This indicates that the standardized ME moderately to highly increases post-test probability to diagnose or exclude common knee disorders.

**Table 4:** Diagnostic validity of the musculoskeletal examination performed by the physiotherapist compared to the physicians’ composite diagnosis

	Sensitivity (95% CI)	Specificity (95% CI)	Positive Likelihood Ratio (95% CI)	Negative Likelihood Ratio (95% CI)
ACL injuries (*n* = 8)	100.0% (52.0–100.0)	99.0% (97.0–100.0)	171.0 (24.2–1207.0)	0.00 (0.00–0.00)
Meniscal injuries (*n* = 36)	97.0% (85.0–100.0)	96.0% (91.0–98.0)	23.2 (10.6–50.8)	0.03 (0.00–0.20)
Patellofemoral pain (*n* = 45)	91.0% (79.0–98.0)	97.0% (93.0–99.0)	30.5 (11.6–80.5)	0.09 (0.04–0.23)
Osteoarthritis (*n* = 79)	91.0% (83.0–96.0)	97.0% (91.0–99.0)	30.4 (10.0–92.8)	0.09 (0.04–0.19)
Others knee disorders (*n* = 11)	82.0% (48.0–98.0)	100.0% (97.0–100.0)	267.6 (16.6–4325.6)	0.18 (0.05–0.64)

*ACL* anterior cruciate ligament. 95% CI: 95% confidence interval

Table 5 presents the concordance between the physiotherapist and the physician for the triage recommendation following consultation. Only 23 participants were considered as surgical candidates and six participants as uncertain by the physicians (see Additional file 2: Appendix 2). Of the 23 patients considered surgical candidates, twenty were evaluated by the orthopaedic surgeons and three by the sports medicine physicians. Among those deemed surgical candidates, five had an ACL tear, seven a meniscal tear and eleven had an OA diagnosis. The overall agreement between the physiotherapist and all physicians was 91.6% with an inter-rater kappa of 0.73 (95% CI: 0.60–0.86). Raw agreement for surgical cases was 91.3% with only two of 23 surgical cases misclassified by the physiotherapist as conservative (see Additional file 2: Appendix 2). Raw agreement for conservative care was 92.6% with 11 of 150 conservative care cases misclassified by the physiotherapist as surgical cases (see Additional file 2: Appendix 2).

**Table 5:** Concordance between the physiotherapist and physicians for the triage recommendation following consultation (*n* = 179)

	Raw agreement	Cohen’s kappa	95% CI
Overall	91.6% (164/179)	0.73	0.60–0.86
Surgical candidates	91.3% (21/23)		
Conservative care candidates	92.6% (139/150)		
Uncertain	66.7% (4/6)		

95% CI: 95% confidence interval

## Discussion

The objectives of our study were to evaluate the diagnostic and surgical triage agreement between a physiotherapist and physicians to assess the validity of the physiotherapist’s musculoskeletal examination (ME) without the use of medical imaging to diagnose common knee disorders. We found high diagnostic agreement and good triage agreement as well as high diagnostic validity for the ME performed by the physiotherapist in patients suffering from common knee disorders and consulting in primary and secondary care settings.

Our results compare well with two previous studies in orthopaedic settings where high inter-rater diagnostic agreement between a physiotherapist and orthopaedic surgeons for the diagnosis of common knee disorders were reported (κ= 0.80 and κ= 0.87 (95% CI: 0.79–0.94)) [8, 32]. Of note, in both these studies, the physiotherapist also had access to imaging results to support his ME which was not the case in our study [8, 32]. Only fourteen patients (*n* = 14) out of 179 (7.8%) were discordant between the physiotherapist using ME compared with the physicians’ composite diagnosis. Discordant patients included one meniscus injury, four patellofemoral pain, seven osteoarthritis one patellar tendinopathy and one functional instability without meniscal or ACL injuries (see Additional file 3: Appendix 3). A possible cause includes a potentially more complex presentation (history and physical examination). Also, because our composite reference standard is done by only one expert, it is possible that the physiotherapist may not be the source of discordance. When comparing the physiotherapist’s diagnosis to imaging diagnoses only, the agreement was somewhat lower supporting the notion that imaging results need to be corroborated with clinical findings from the ME and that these findings may be more important to make a diagnosis [32].

Another objective of our study was to evaluate the diagnostic validity of a ME performed without imaging support. This objective is important in the context where the need to rely on imaging may delay care either by the physiotherapist or a physician or the ordering of a given imaging may be altogether unnecessary to make a diagnosis and initiate appropriate care. Our results show that a ME performed by a physiotherapist without imaging could reach moderate to high diagnostic validity to diagnose or exclude common knee disorders and these results are comparable to already published evidence. *Jackson* et al. reported in their meta-analysis LR+≥ 10 for lateral meniscus, ACL injuries and cartilage lesions and LR-≤ 0.20 for lateral and medial meniscus and LR- = 0.27 for ACL injury confirming the validity of a complete clinical examination performed by orthopaedic surgeons or sports medicine physicians [1]. What remains to be established is what is the optimal combination of history questions and physical examination tests results in the ME that is helpful to support the differential diagnosis of common knee disorders [39–43]. Nonetheless, the use of imaging may be warranted in more complex cases or to diagnose or exclude uncommon disorders when the expected recovery after initiation of care is not as predicted. In this situation, it is interesting to see that published evidence support physiotherapists to refer patients autonomously and appropriately to imaging [5, 8].

Our findings regarding concordance for triage recommendations of surgical candidates are consistent with three previous studies demonstrating good triage agreement with an orthopaedic surgeon (raw agreement: 87% and 91.8%; κ=0.77; 95%CI: 0.65–0.88, respectively) [8, 32, 44]. As stated above, the physiotherapist had access only to the ME without imaging to make the triage recommendation. Recent evidence proposes that pain, functional limitations and clinical symptoms should be used as surgical eligibility criteria for ACL injuries [45], meniscal injuries [46, 47] and knee OA [48, 49] and not systematically rely on imaging results. In our study, 82% (116/142) of secondary care participants and 92% (34/37) of primary care participants were referred to conservative care after their first consultation. Almost all of these patients (92.6%) would have been appropriately triaged to conservative care directly by the physiotherapist based only on the ME, making their care trajectory likely more efficient. Interestingly, this suggests that our cohort spectrum and representativeness is balanced between primary care and a pure secondary or tertiary surgical setting. Therefore, a well-executed ME may provide appropriate findings to guide patients to the appropriate care and these results suggests the role of physiotherapists as qualified musculoskeletal experts for knee disorders [44, 50].

### Strengths and limitations

Our prospective cohort was recruited from three different settings, both in primary and secondary care, allowing for a broad variety of patients with various knee disorders, thus limiting spectrum bias commonly encountered in other diagnostic studies [51]. However, most patients were recruited in orthopedic clinics and this may limit the applicability of the findings to other settings. Evaluators met prior to the initiation of the study to standardize techniques and interpretation of the physical tests and related diagnoses, but no formal evaluation of their skills was undertaken. Also, the physiotherapist always evaluated the patients prior to the physician and this may have increased the diagnostic concordance by sensitizing the patients even though evaluators remained blinded to each others results. Our composite reference standard included both musculoskeletal examination and imaging interpretation by experienced medical experts, which is considered clinically relevant in the study of musculoskeletal disorders [24–27]. However, only one physiotherapist and one of four medical experts evaluated each given participant and this limits the generalizability of our results. It must be noted that the physiotherapist in our study had only one year of clinical experience, which is suggestive of the appropriateness of physiotherapy training programs to adequately train therapists in musculoskeletal examination, but will require confirmation with more physiotherapists of diverse level of experience.

## Conclusions

High diagnostic agreement and good triage of surgical candidates agreement was found between the physiotherapist and experienced physicians for various knee problems. Musculoskeletal examination without imaging performed by trained musculoskeletal providers may yield high diagnostic validity to discriminate between common knee disorders. This suggests the potential role of healthcare professionals such as physiotherapists in the development of multidisciplinary evaluation and triage strategies in the context of innovative and potentially more efficient care trajectories for patients with common musculoskeletal disorders. These results will require confirmation with a larger study, in other primary care settings, with a greater number of physiotherapists and for other musculoskeletal disorders.

## Additional files

Additional file 1: Appendix 1. Flowchart of patients recruitment. (DOCX 59 kb)

Additional file 2: Appendix 2. 3 × 3 table for triage of surgical candidates and conservative care. (DOCX 43 kb)

Additional file 3: Appendix 3. 2 × 2 tables for diagnoses of knee disorders. (DOCX 83 kb)

## Abbreviations

ACL: Anterior cruciate ligament
LR: Likelihood ratio
NPV: Negative predictive value
OA: Osteoarthritis
PFP: Patellofemoral pain
PPV: Positive predictive value
Se: Sensitivity
Sp: Specificity
