# Supplementary material for: Diagnostic validity and triage concordance of a physiotherapist compared to physicians’ diagnoses for common knee disorders
Source: BMC Musculoskelet Disord. 2017 Nov 14;18:445. doi: 10.1186/s12891-017-1799-3 (PMC5686957; doi:10.1186/s12891-017-1799-3)
Supplement: Supplementary file 1 — Flowchart of patients recruitment. (DOCX 59 kb) [file 12891_2017_1799_MOESM1_ESM.docx]

**APPENDIX 1: Flowchart of patients recruitment**

Patients recruited

n=198

Refused to participate

n=5 (2.5%)

*Lack of time (n=3)*

*Not interested (n=2)*

Patients included

n=179 (90.4%)

Excluded before consultation

n=14 (7.1%)

*Trauma on total knee arthroplasty (n=1)*

*Prior visit with one of the participating physicians (n=4)*

*Systemic rheumatology disorders (n=2)*

*Under 18 years old (n=1)*

*Do not understand French (n=3)*

*Not able to consent, mental health disorders (n=3)*
