# Supplementary material for: Diagnostic validity and triage concordance of a physiotherapist compared to physicians’ diagnoses for common knee disorders
Source: BMC Musculoskelet Disord. 2017 Nov 14;18:445. doi: 10.1186/s12891-017-1799-3 (PMC5686957; doi:10.1186/s12891-017-1799-3)
Supplement: Supplementary file 2 — 3 × 3 table for triage of surgical candidates and conservative care. (DOCX 43 kb) [file 12891_2017_1799_MOESM2_ESM.docx]

**APPENDIX 2: 3x3 table for triage of surgical candidates and conservative care**

|  |  | | | | | |
| --- | --- | --- | --- | --- | --- | --- |
|  | | | Medical expert | | | |
|  |  |  | Yes | No | Uncertain | Total |
| Physiotherapist | | Yes | 21 | 11 | 0 | 32 |
|  |  | No | 2 | 139 | 2 | 143 |
|  |  | Uncertain | 0 | 0 | 4 | 4 |
|  | | Total | 23 | 150 | 6 | 179 |

“Yes” are surgical candidates, “No” are conservative care candidates, “Uncertain” needed more information to confirm treatment options.
