# Supplementary material for: Diagnostic validity and triage concordance of a physiotherapist compared to physicians’ diagnoses for common knee disorders
Source: BMC Musculoskelet Disord. 2017 Nov 14;18:445. doi: 10.1186/s12891-017-1799-3 (PMC5686957; doi:10.1186/s12891-017-1799-3)
Supplement: Supplementary file 3 — 2 × 2 tables for diagnoses of knee disorders. (DOCX 83 kb) [file 12891_2017_1799_MOESM3_ESM.docx]

**APPENDIX 3: 2x2 tables for diagnoses of knee disorders**

|  | | | | |
| --- | --- | --- | --- | --- |
| **ACL injury** | | Medical expert | | |
|  |  | Yes | No | Total |
| Physiotherapist | Yes | 8 | 1 | 9 |
|  | No | 0 | 170 | 170 |
|  | Total | 8 | 171 | 179 |

| **Meniscal injury** | | Medical expert | | |
| --- | --- | --- | --- | --- |
|  |  | Yes | No | Total |
| Physiotherapist | Yes | 35 | 6 | 41 |
|  | No | 1 | 137 | 138 |
|  | Total | 36 | 143 | 179 |

| **Patellofemoral pain** | | Medical expert | | |
| --- | --- | --- | --- | --- |
|  |  | Yes | No | Total |
| Physiotherapist | Yes | 41 | 4 | 45 |
|  | No | 4 | 130 | 134 |
|  | Total | 45 | 134 | 179 |

| **Osteoarthritis** | | Medical expert | | |
| --- | --- | --- | --- | --- |
|  |  | Yes | No | Total |
| Physiotherapist | Yes | 72 | 3 | 75 |
|  | No | 7 | 97 | 104 |
|  | Total | 79 | 100 | 179 |

| **Others knee disorder*** | | Medical expert | | |
| --- | --- | --- | --- | --- |
|  |  | Yes | No | Total |
| Physiotherapist | Yes | 9 | 0 | 9 |
|  | No | 2 | 168 | 170 |
|  | Total | 11 | 168 | 179 |

**Other diagnosis included: contusion of the tibial plateau (n=2), PCL tear (n=1), soleus tear (n=1), psychosomatic origin (n=1), muscular spasms linked to multiple sclerosis (n=1), hamstring tendinopathy (n=3), medial collateral ligament injury (n=1), functional instability in the absence of ACL or meniscal injury (n=1).*
